# Effect of antecolic or retrocolic reconstruction of the gastro/duodenojejunostomy on delayed gastric emptying after pancreaticoduodenectomy: a meta-analysis

**DOI:** 10.1186/s12876-015-0300-8

**Published:** 2015-06-16

**Authors:** Yanming Zhou, Jincan Lin, Lupeng Wu, Bin Li, Hua Li

**Affiliations:** 1 Department of Hepatobiliary & Pancreatovascular Surgery, First affiliated Hospital of Xiamen University; Oncologic Center of Xiamen, Xiamen, China; 2 Department of Digestive Diseases, First Xiamen Hospital, Fujian Medical University, Xiamen, China

**Keywords:** Delayed gastric emptying, Gastro/duodenojejunostomy reconstruction, Pancreatoduodenectomy, Meta-analysis

## Abstract

**Background:**

Delayed gastric emptying (DGE) is one of the most frequent complications after pancreaticoduodenectomy (PD). This meta-analysis aimed to evaluate the effect of antecolic versus retrocolic reconstruction of gastro/duodenojejunostomy on DGE after PD.

**Methods:**

Randomized controlled trials (RCTs) comparing antecolic versus retrocolic reconstruction of gastro/duodenojejunostomy on DGE after PD were eligible for inclusion. Pooled estimates of treatment effect were calculated using either the fixed effects model or random effects model.

**Results:**

Five RCTs involving 534 randomized patients were eligible. The comparison of DGE showed no significant difference (odds ratio, 0.66; 95 % confidence interval, 0.32 to 1.33; *P* = 0.24). The antecolic and retrocolic groups also had comparable outcomes for clinical parameters related to DGE, other complications, hospital mortality, and length of hospital stay.

**Conclusions:**

The route of gastro/duodenojejunostomy reconstruction has no impact on DGE after PD. Therefore, the choice of reconstruction route should be selected according to the surgeon’s preference.

## Background

With the refinements in surgical techniques, improvements in perioperative management, advancements in surgical instruments, pancreaticoduodenectomy (PD) has become a safer procedure with a reported operative mortality less than 5 % at high-volume centres. However, the incidence of morbidity approaches 30–65 % [1]. Delayed gastric emptying (DGE) is one of the most frequent morbidity after PD occurring in 19–57 % of patients [2]. It has been associated with longer duration of hospitalization and higher hospital costs.

Two reconstruction routes are usually used for gastro/duodenojejunostomy: the antecolic route or the retrocolic route. A meta-analysis published by Su et al [3] compared 5 studies [4–8] and concluded that antecolic reconstruction route was associated with a statistically significant decrease in the incidence of DGE following PD. However, this meta-analysis included three observational studies [4–6], which may introduce confounding and selection bias that often distort the findings. The randomised controlled trial (RCT) is the principal research design in the evaluation of medical interventions and is best confined to meta-analysis [9]. More recently, four RCTs have become available and reported that the route of gastro/duodenojejunostomy reconstruction does not influence the postoperative incidence of DGE or other complications after PD [10–13]. Therefore, the present meta-analysis provides an updated evaluation by pooling data that only come from the RCTs.

## Methods

The study was conducted following the Preferred Reporting Items for Systematic Reviews and Meta- Analyses (PRISMA) [14].

### Study selection

Using Medline, EMBASE, OVID, and Cochrane database, a literature search was made for RCTs that evaluated the influence of an antecolic with a retrocolic gastro/duodenojejunostomy reconstruction on DGE after PD from the time of inception to November 2013. The Medical Subject Heading (MeSH) search terms were “pancreaticoduodenectomy” and “delayed gastric emptying.” Only studies on humans and in the English language were considered for inclusion. Reference lists of all retrieved articles were manually searched for additional studies.

### Data extraction

Two reviewers (B.L. and L.W., respectively) independently extracted the following parameters from each study: first author, year of publication, study population characteristics, number of patients randomized with each procedure, and endpoints. All relevant text, tables and figures were reviewed for data extraction.

#### Criteria for inclusion and exclusion

RCTs that evaluated the influence of an antecolic with a retrocolic gastro/duodenojejunostomy reconstruction on DGE after PD were included in the study. Exclusion criteria were: animal studies, abstracts, letters, proceedings from scientific meetings, editorials and expert opinions, and non-randomized observational clinical studies.

### Assessment of methodological quality

The RCTs were scored using the Jadad composite scale [15] in which each study was evaluated by examining 3 factors: randomization, blinding, and withdrawals and drop-outs reported within the study period. The quality scale ranges from 0 to 5 points, study having 3 or more score was considered to be of higher quality.

### Endpoints

Primary endpoint was DGE. Secondary endpoints included other complications and length of hospital stay.

### Statistical methods

Review Manager (RevMan) software 5.0 (Cochrane Collaboration) was used to conduct all analyses. Estimated effect measures were odds ratios (OR) for dichotomous variables and weighted mean difference (WMD) for continuous variables. If the study provided medians and interquartile ranges instead of means and SDs, the means and SDs were imputed according to the methods described by Hozo et al. [16] Pooled estimates were presented with 95 % confidence intervals (95 % CI). Pooled effect was calculated using either the fixed effects model or random effects model. Heterogeneity was evaluated by I^2^, with values over 50 % indicating considerable heterogeneity. Publication bias was assessed visually using a funnel plot, based on the result of DGE.

## Results

### Eligible studies

**Fig. 1:**
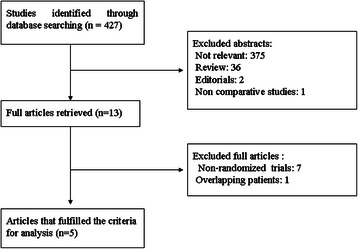
Study flow chart

The process of identifying eligible literatures is shown in Fig. 1. The search strategy generated 6 RCTs. Two studies from the same group [8, 11], the most recent study that including more subjects was selected [11]. Finally, five articles were identified for inclusion [7, 10–13]. The two reviewers had 100 % agreement in their reviews of the data extraction.

A total of 534 patients were included in the meta-analysis: 267 in the antecolic group and 267 in the retrocolic group. Two studies were conducted in Japan [7, 11], one in India [10], one in the Netherlands [12], and one in Austria [13]. The sample size of each study varied from 40 to 246 patients. The characteristics of the included studies are shown in Table 1.

**Table 1:** Baseline characteristics of studies included in the meta-analysis

Reference (Year)	Enrolment interval (country)	Group	No. of patients (M /F)	Mean age (years)	Disease Ma/Be	Type of operation	Quality score
Tani et al. [7] (2006)	2002-2004 (Japan)	Antecolic	20 (11/9)	63.1 ± 9.21	16/4	All PPPD	2
		Retrocolic	20 (10/10)	66.7 ± 12.2	16/4	All PPPD	
Gangavatiker et al. [10] (2011)	2006-2008 (India)	Antecolic	32 (23/9)	52.8 ± 11.6	27/5	PPPD:10; CPD:22	2
		Retrocolic	36 (26/10)	50.8 ± 10.6	32/4	PPPD:14; CPD:22	
Imamura et al. [11] (2013)	2005-2011 (Japan)	Antecolic	58 (36/22)	70.0 (36–86)	46/12	All PPPD	2
		Retrocolic	58 (32/26)	69.0 (46–86)	49/9	All PPPD	
Eshuis et al. [12] (2013)	2009-2011 (the Netherlands)	Antecolic	121 (83/38)	65.4 ± 9.0	108/13	PPPD:93; CPD:28	2
		Retrocolic	125 (68/57)	65.2 ± 10.3	119/6	PPPD:105; CPD:20	
Tamandl et al. [13] (2013)	2007-2009 (Austria)	Antecolic	36 (17/19)	67.1 (55.7–75.3)	28/8	All PPPD	2
		Retrocolic	28 (12/16)	65.4 (55.6–70.6)	20/8	All PPPD	

M /F, Male/Female; Ma/Be, Malignant/benign; PPPD, pylorus-preserving pancreaticoduodenectomy; CPD, classic pancreaticoduodenectomy

### Outcomes assessed

Table 2 shows the results for the outcomes.

**Table 2:** Results of a meta-analysis

Outcome of interest	No. of studies	No.of patients	OR/WMD	95 % CI	*P*-value	I^2^ (%)
DGE	5	534	0.66	0.32, 1.33	0.24	57
ISGPS DGE	3	430	0.97	0.64, 1.47	0.89	0
ISGPS B + C DGE	3	430	0.93	0.60, 1.46	0.76	0
Removal of NGT (day)	5	534	0.28	-0.30, 1.06	0.27	72
Reinsertion of NGT	4	494	1.14	0.73, 1.81	0.56	31
Prokinetics or anti/emetics	2	314	0.84	0.53, 1.32	0.45	0
Start of liquid diet (day)	2	184	0.26	-0.63, 1.16	0.56	0
Start of solid diet (day)	4	470	-0.90	-1.91, 0.10	0.08	0
Pancreatic fistula	5	534	1.05	0.69, 1.61	0.80	0
Intra-abdominal abscess	5	534	1.04	0.62, 1.75	0.88	8
Hemorrhage	5	534	0.74	0.37, 1.48	0.40	0
Bile leak	5	534	1.09	0.48, 2.51	0.83	15
Wound infection	5	534	0.92	0.60, 1.40	0.70	0
Reoperation	3	354	0.49	0.22, 1.09	0.08	0
Mortality	3	350	0.60	0.22, 1.64	0.32	0
Length of hospital stay (days)	5	534	0.44	-0.30, 1.17	0.25	26

DGE, delayed gastric emptying; ISGPS, International Study Groups of Pancreatic Surgery; NGT, nasogastric tube

OR, odds ratio; WMD, weighted mean difference; CI, confidence interval

**Fig. 2:**
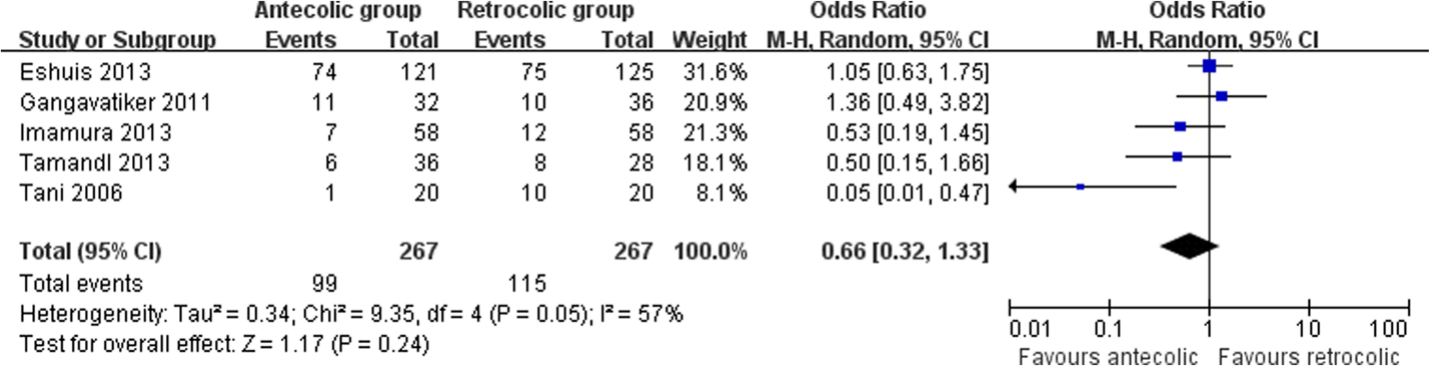
Results of the meta-analysis on delayed gastric emptying

All studies provided information on the incidence of DGE, which occurred in 37.1 % of patients in the antecolic group versus 43.1 % of patients in the retrocolic group. Polled analysis showed that there was no significant difference between groups (OR 0.66, 95 % CI, 0.32 to 1.33; *P* = 0.24). Considerable heterogeneity was detected between studies (I^2^ = 57 %) (Fig. 2). In three studies [10–12], DGE was defined and graded according to the recommendations of the International Study Group of Pancreatic Surgery (ISGPS) [17]. Pooled analysis showed both overall DGE (OR 0.97, 95 % CI, 0.64 to 1.47; *P* = 0.89) and clinically significant DGE (grade B or C) (OR 0.93, 95 % CI, 0.60 to 1.46 *P* = 0.76) were not different with no significant heterogeneity.

Measures of the clinical parameters related to DGE were all comparable between groups: namely, time of removal of nasogastric tube (WMD 0.38, 95 % CI, -0.30 to 1.06; *P* = 0.27), requirement for reinsertion of nasogastric tube (OR 1.14, 95 % CI, 0.73 to 1.81; *P* = 0.56), requirement of prokinetics or anti/emetics(OR 0.84, 95 % CI, 0.53 to 1.32; *P* = 0.45), time of start of liquid diet (WMD 0.26, 95 % CI, -0.63 to 1.16; *P* = 0.56), and time of start of solid diet (WMD -0.90, 95 % CI, -1.91 to 0.10; *P* = 0.08). No significant heterogeneity was found between studies regarding these outcomes, except for the time of removal of nasogastric tube .

**Fig. 3:**
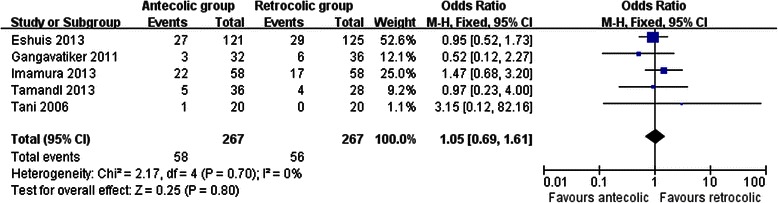
Results of the meta-analysis on pancreatic fistula

**Fig. 4:**
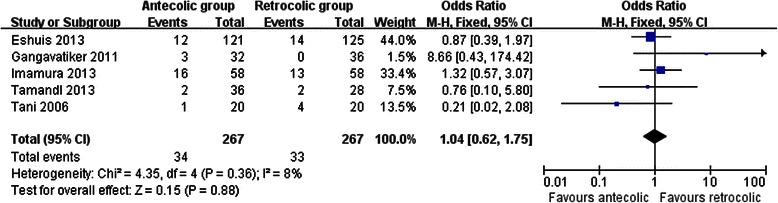
Results of the meta-analysis on intra-abdominal abscess

**Fig. 5:**
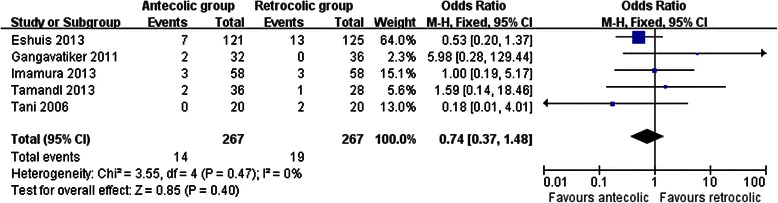
Results of the meta-analysis on hemorrhage

**Fig. 6:**
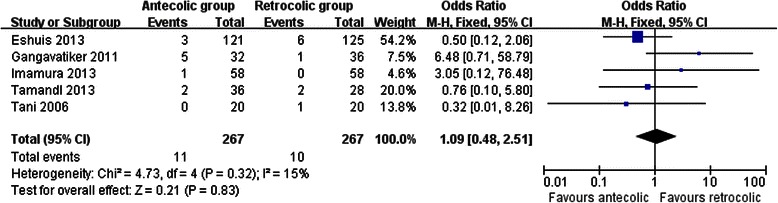
Results of the meta-analysis on bile leakage

**Fig. 7:**
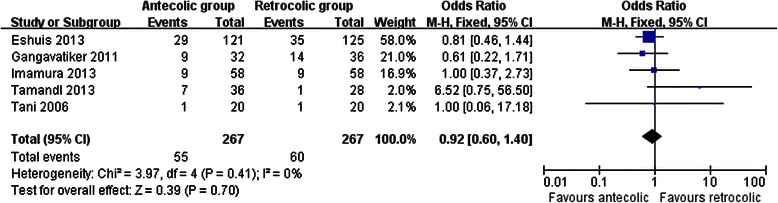
Results of the meta-analysis on wound infection

**Fig. 8:**
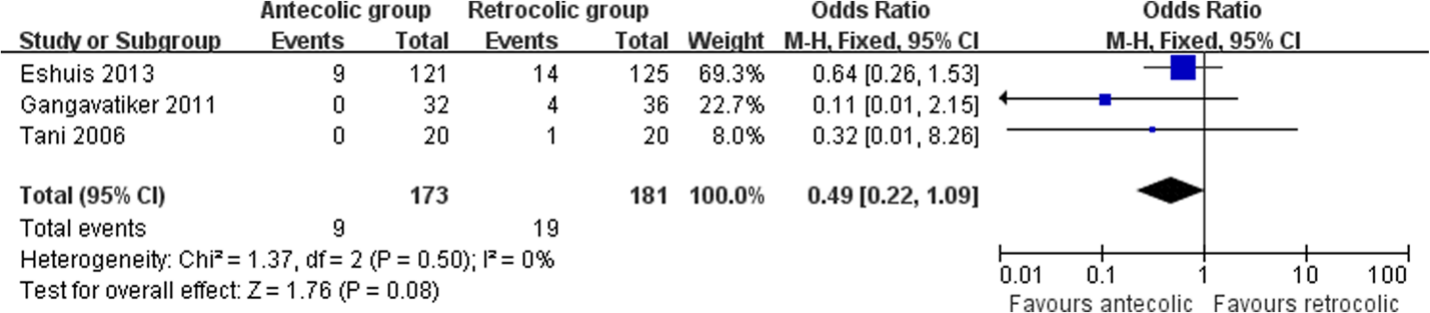
Results of the meta-analysis on reoperation

**Fig. 9:**
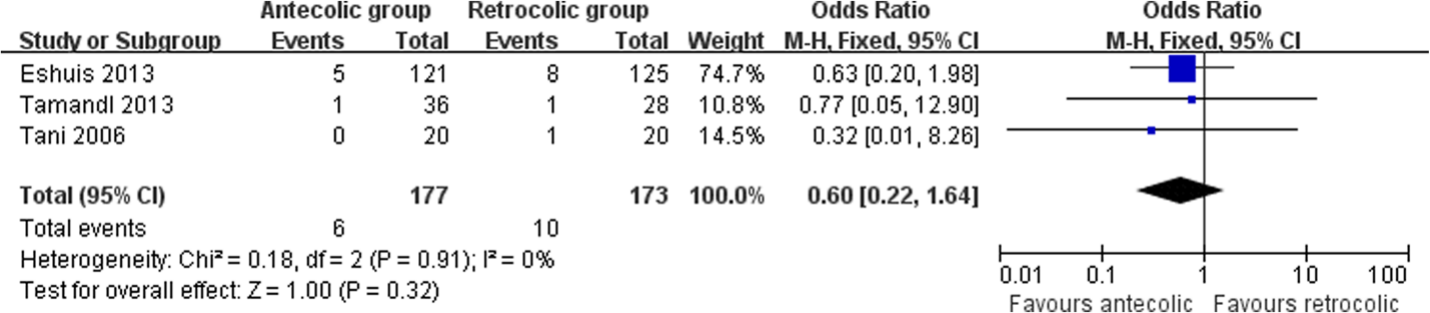
Results of the meta-analysis on hospital mortality

**Fig. 10:**
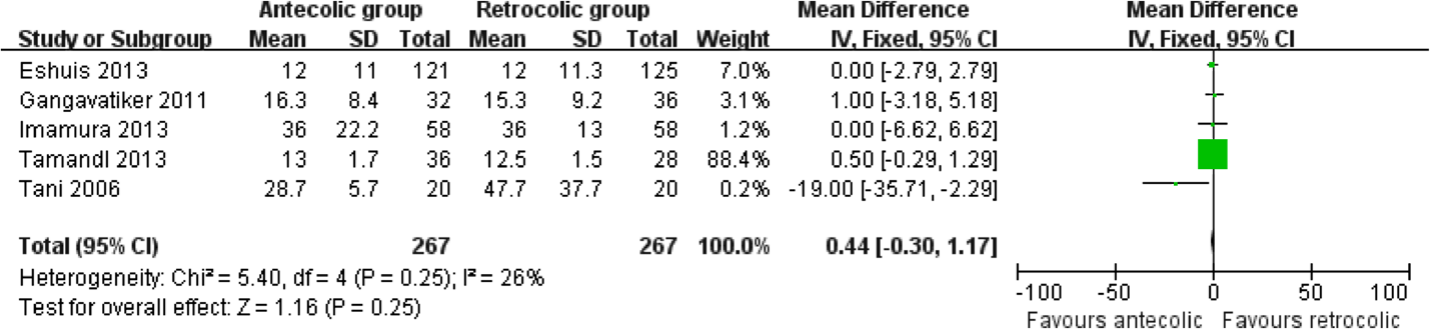
Results of the meta-analysis on length of hospital stay

Measures of secondary endpoints were also not significantly different between the two groups: namely, pancreatic fistula (OR 1.05, 95 % CI, 0.69 to 1.61; *P* = 0.80) (Fig. 3), intra-abdominal abscess (OR 1.04, 95 % CI, 0.62 to 1.75; *P* = 0.88) (Fig. 4), hemorrhage (OR 0.74, 95 % CI, 0.37 to 1.48; *P* = 0.40) (Fig. 5), bile leakage (OR 1.09, 95 % CI, 0.48 to 2.51; *P* = 0.83) (Fig. 6), wound infection (OR 0.92, 95 % CI, 0.60 to 1.40; *P* = 0.70) (Fig. 7), reoperation (OR 0.49, 95 % CI, 0.22 to 1.09; *P* = 0.08) (Fig. 8), hospital mortality (OR 0.60, (95 % CI, 0.22 to 1.64; *P* = 0.32) (Fig. 9), and length of hospital stay (WMD 0.44, 95 % CI, -0.30 to 1.17; *P* = 0.25) (Fig. 10). No significant heterogeneity was found between studies regarding these outcomes.

#### Publication bias

**Fig. 11:**
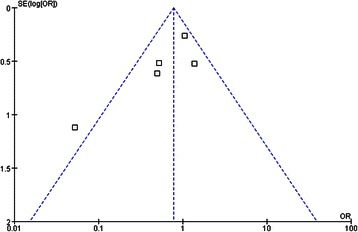
Funnel plot analysis of publication bias. The outcome was the delayed gastric emptying

The funnel plot for the primary outcome (DGE) was asymmetric, indicating the presence of publication bias (Fig. 11).

## Discussion

DGE after PD is a frequent complication, which is usually managed by nasogastric drainage and nutritional support by parenteral or enteral routes, with or without prokinetics. Many efforts for reducing the incidence of DGE have been attempted; these include pyloric dilation [18], preservation of the left gastric vein [19], preoperative use of erythromycin [20], and prophylactic octreotide [21]. Other studies evaluated the clinical efficacy of reconstruction procedure of gastric emptying. As compared with Billroth II reconstruction, both Roux-en-Y and Billroth-I reconstructions were found to be associated with higher incidence of DGE [22, 23]. Two routes are usually used for Billroth II reconstruction after PD: the antecolic route or the retrocolic route. A meta-analysis reported the superiority of the antecolic route compared with the retrocolic route concerning the reduction of DGE [3]. However, a significant proportion of data in this meta-analysis came from nonrandomized studies, which may introduce confounding and selection bias that often distort the findings.

The present updated meta-analysis pooled five RCTs and provided clearly the best available evidence on the effect of reconstruction route concerning DGE. In contrast with previously published meta-analysis, the main finding is that the two routes after PD were equally efficient concerning DGE. There is wide variation definition of a DGE in the pancreatic surgery literature. In 2007, the ISGPS proposed a standardized definition of DGE [17]. Three of 5 RCTs used the ISGPS criteria and consistently found that the route of gastro/duodenojejunostomy reconstruction had no significant impact on the incidence and severity of DGE. The pooled data is also in concordance with these RCTs.

The pathogenesis of DGE after pancreatoduodenectomy has been proposed to be multifactorial: disruption of the vagal nerve system; ischemic injury to the antropyloric mechanism; and decreased plasma motilin stimulation caused by resection of the duodenum [20]. From a mechanical point of view, some researchers observed that a transient torsion or angulation of the reconstructed alimentary tract might contribute to DGE [24]. With antecolic reconstruction, the duodenal stump or distal stomach and the descending jejunal loop are set in a straight line. Torsion or angulation of the reconstructed alimentary tract can thus be avoided [25]. However, in case of retrocolic reconstruction, the risk of torsion or angulation can be diminished by suturing the duodenum or distal stomach to the transverse mesocolon [12]. Thus, one can understand that the reconstruction route has no measurable impact on the incidence and severity of DGE.

Regarding the operative technique, it has been suggested that DGE more likely occurs in patients who underwent pylorus-preserving pancreaticoduodenectomy (PPPD) (in comparison with classic Whipple PD). However, a recent meta-analysis of six RCTs showed an overall comparable rate of DGE for both techniques. [26] Also, the type of pancreatic anastomosis (pancreaticogastrostomy and pancreaticojejunostomy) was not significantly associated with DGE [27]. By contrast, there are growing evidences that other intraabdominal complications, such as pancreatic fistula, biliary fistula, intraabdominal collections or abscesses, have a critically influence on DGE. Park et al [28] found that DGE was significantly more frequent among patients with postoperative intraabdominal complications (41.7 % versus 8.8 %; *P<*0.0001). Similarly, in another report by Horstmann et al [29], DGE almost exclusively occurs as a consequence of other postoperative complications. These findings are supported by those of other reports [21, 30]. Hence, prevention of such complications might reduce the incidence of DGE.

This present analysis has some limitations. First, considerable heterogeneity was detected between studies regarding primary endpoint. The presence of heterogeneity is due to paper by Tani et al [7] in which incidence of DGE of 5 % in their antecolic group compared to 50 % in the retrocolic group (*P<*0.001). Apart from the fact that there were only 20 patients in each arm in this study, a total of 12/20 patients in the retrocolic group had at least one postoperative complication as compared to 3/20 in the antecolic group might have influenced their results [10]. Second, the number of studies included in this meta-analysis is small. Indeed, randomised trials in surgery are difficult to conduct [31]. Finally, funnel plot analysis suggested the possibility of publication biases. This may relate to our inclusion of English only studies.

## Conclusions

Our meta-analysis did not observe a significant effect of the kind of reconstruction route on the incidence of DGE after PD. Moreover, we did not find any differences in terms of hospital stay, other complications, and mortality between two groups, underlining the safety of both procedures. Therefore, the choice of reconstruction route should be selected according to the surgeon’s preference.
